# Research of Vertical via Based on Silicon, Ceramic and Glass

**DOI:** 10.3390/mi14071391

**Published:** 2023-07-08

**Authors:** Wenchao Tian, Sixian Wu, Wenhua Li

**Affiliations:** School of Electro-Mechanical Engineering, Xidian University, Xi’an 710000, China

**Keywords:** TSV, TGV, TCV, process optimization

## Abstract

With the increasing demand for high-density integration, low power consumption and high bandwidth, creating more sophisticated interconnection technologies is becoming increasingly crucial. Three-dimensional (3D) integration technology is known as the fourth-generation packaging technology beyond Moore’s Law because of its advantages of low energy consumption, lightweight and high performance. Through-silicon via (TSV) is considered to be at the core of 3D integration because of its excellent electrical performance, lower power consumption, wider bandwidth, higher density, smaller overall size and lighter weight. Therefore, the particular emphasis of this review is the process flow of TSV technology. Among them, the research status of TSV hole etching, deep hole electroplating filling and chemical mechanical planarization (CMP) in TSV preparation process are introduced in detail. There are a multitude of inevitable defects in the process of TSV processing; thus, the stress problems and electrical characteristics that affect the reliability of TSV are summarized in this review. In addition, the process flow and process optimization status of through ceramic via (TCV) and through glass via (TGV) are discussed.

## 1. Introduction

**Figure 1:**
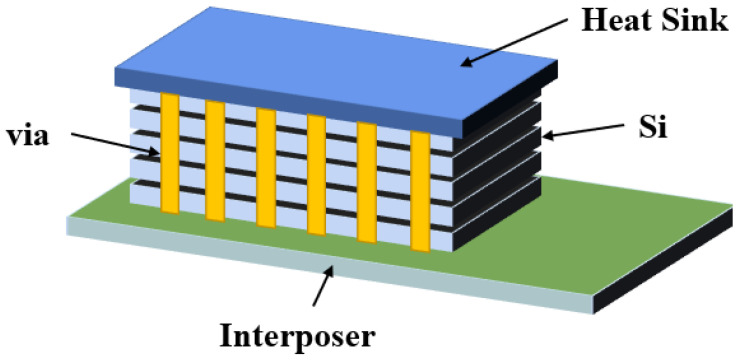
Schematic diagram of a simple 3D integrated circuit.

Three-dimensional (3D) integration has the advantages of low power consumption, small form factor, high performance and high functional density [1,2,3,4]. It can meet the interconnection requirements of low latency and low power consumption by shortening the interconnection length [5,6,7,8]. A circuit diagram is shown in Figure 1 [9]. As early as 2010, the European Union launched the Best-Reliable Ambient Intelligent Nano Sensor Systems (E-BRAIN) and carried out research on 3D micro-system integration technology with 3D vertical interconnection as its core, with the aim to develop reliable and high-yield wafer-level 3D stacked integration technology [10]. Among them, through-silicon via (TSV) technology has become the key to the development of 3D packaging technology [11]. TSV technology refers to the processing of micro-vias on the silicon wafer and filling conductive materials in the silicon wafer. Through TSV technology, vertical interconnection between chips, integration and data transmission between homogeneous or heterogeneous chips can be realized, which can significantly improve device performance and the integration of a single device. Compared with traditional lateral interconnects, TSV structures have the shortest path in the vertical direction; this characteristic not only shortens the signal transmission path, reduces the transmission resistance and reduces the generation of heat but also improves the high-frequency performance of the chip. On the other hand, this vertical structure effectively reduces the area of interconnect structures on the chip. Under the same area, the stack structure formed by TSV has higher performance and more functionality [12]. TSV is not only widely used in the field of information technology but also has potential in the design and production of future mainstream devices [13]. In view of the wide application of stereoscopic integration in the dual-use market, China has strongly supported stereoscopic integration technology based on TSV [14]. In 2011, China established the TSV technology research consortium to vigorously promote the industrialization of civil TSV technology. At present, the main TSV research units carry out “via-last” technology research. The research on TSV 3D integration has been supported by basic scientific research, microelectronics, sophisticated manufacturing, and very large-scale integrated circuit production technologies [15]. A study [16] conducted by French consulting firm Yole Development found that TSV can be applied to almost any chip package and any type of advanced package, including light emitting diodes (LED) and micro-electro-mechanical systems (MEMS). It was reported by the Fraunhofer Institute in Germany that inertial MEMS chips and integrated circuit (IC) chips with corresponding functions can be assembled on the 2.5D TSV adapter board by using the processes of redistribution layer (RDL), micro bump and flip chip bonding, which provides a technical approach for 3D integration of inertial micro-system and a design architecture space for integrating more functional chips.

**Figure 2:**
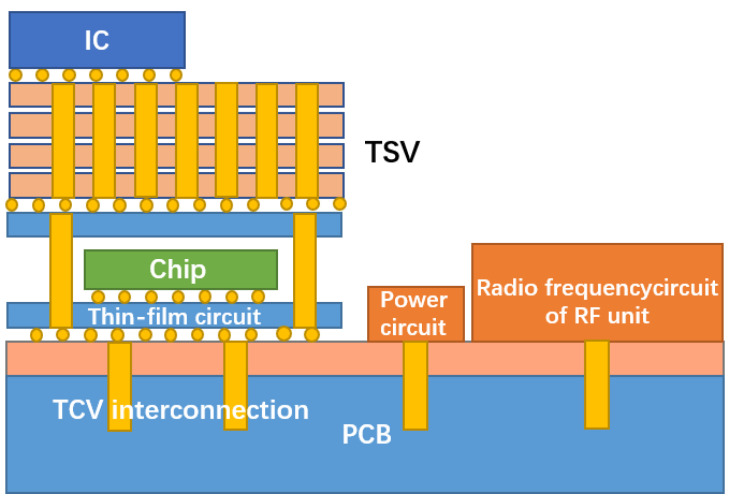
Schematic diagram of a TCV integrated structure.

With the requirements of miniaturization, lightweight, high integration, high reliability and the low cost of electronic equipment, microwave circuits urgently need to stack microwave multi-chip modules with different functions in the *Z*-axis direction to form a vertically interconnected 3D multi-chip module [17]. Through ceramic via (TCV) technology expands the volume of high-precision and high-power thin-film circuits with 3D planar distribution, significantly improves the structural density and reduces the device size through via interconnection and circuit redistribution. Components based on TCV technology have a wide application prospect in micro and small product systems. The physical architecture model of TCV is shown in Figure 2 [18].

**Figure 3:**
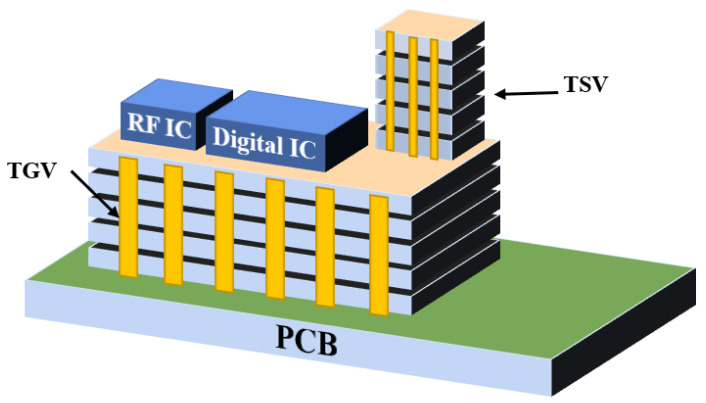
Schematic diagram of a system-level package based on a glass adapter plate.

In recent years, 3D integrated packaging has become the main direction of advanced packaging development, and most 3D integration uses silicon as the substrate. However, research institutions, such as the Georgia Institute of Technology, have used glass as the substrate to realize 3D integration. Figure 3 is a schematic diagram of the system-in-package based on a glass transfer plate described by the Georgia Institute of Technology [19]. Through glass via (TGV) technology has the advantages of high-density interconnection and low-loss transmission, which forms a highly reliable interconnection between the chip and the substrate, has higher I/O density and smaller spacing, and realizes passive on-chip devices, high-density copper interconnection and the heterogeneous integration of chips. Therefore, 3D integration and system-level packaging technology have developed rapidly [20]. By utilizing TGV technology and new design technology, it is possible to build miniaturized new devices and integrated 3D radio frequency (RF) microsystems.

## 2. TSV Technology Process and Development

TSV is the latest interconnection scheme to realize signal transmission by stacking chips vertically and connecting layers vertically [21,22]. In order to realize signal transmission, a metal with good conductivity is filled in the center of the through hole to realize the interconnection. At the same time, in order to avoid signal leakage to the silicon substrate through the metal layer, an insulating layer is often added between the metal and the silicon substrate to minimize signal loss. Through the use of silicon vias, the integration of chips is further improved, which avoids the idleness and waste of space, thus improving the stacking density of chips. At the same time, due to the vertical spatial interconnection, the transmission efficiency and reliability of signals are greatly improved [23]. The application of silicon vias makes it possible to integrate, miniaturize and reduce the power consumption of chips, which is the most promising interconnection technology in the field of electronic packaging at present. Before using silicon vias, integrated chips often use a combination of metal wiring and wire bonding technology to realize the interconnection packaging. This method has the disadvantage of long signal transmission distance and significant signal loss, which reduces the reliability of the channels and circuits. Additionally, the interconnection wiring in the plane layer is complicated, which can lead to mutual interference between the signals and some devices. Furthermore, the plane wiring also occupies a certain use area of the chip [24].

### 2.1. Silicon through Hole Manufacturing Process

**Figure 4:**
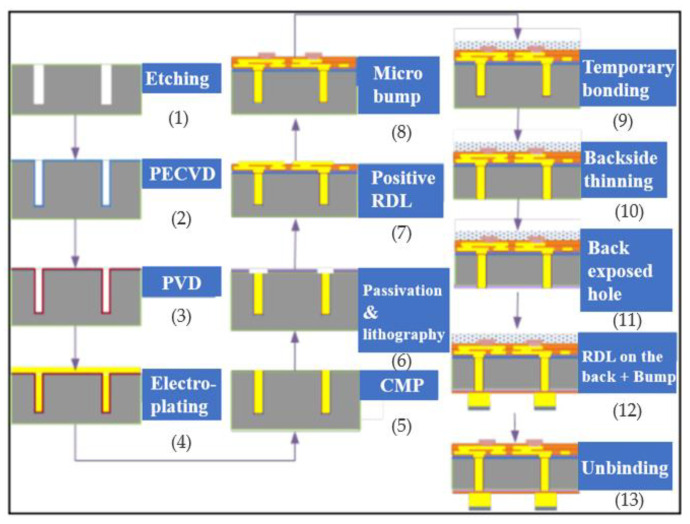
Flowchart of the silicon substrate manufacturing process (Reprinted with permission from Ref. [10], 2021, Wu, D.-W.).

Taking cylindrical Cu-TSV as an example, the manufacturing process is described here. The TSV process flow chart is shown in Figure 4 [10]; process (1) is to form silicon through holes by deep silicon etching; process (3) is to deposit and make an insulating layer, barrier layer and seed layer by physical deposition; then, choosing an electroplating method to fill the blind hole with copper; process (4) is to use chemical and mechanical polishing methods to remove excess copper. Once the copper filling is completed, the wafer needs to be thinned in process (7); Finally, the process (9) is wafer bonding. The following is a detailed introduction to the main processes.

#### 2.1.1. Deep Silicon Etching

**Figure 5:**
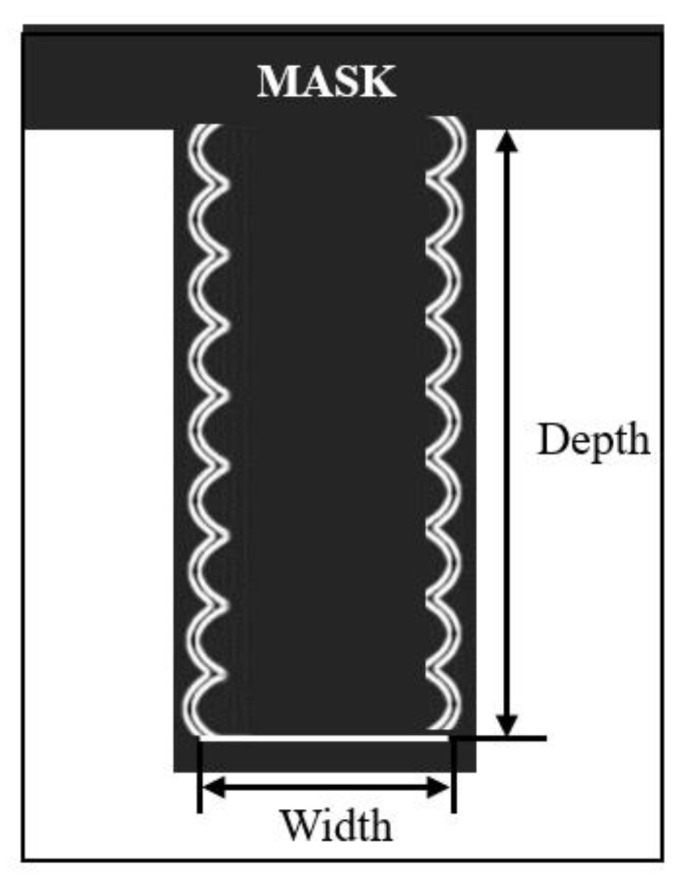
Schematic diagram of the silicon deep groove etching in the Bosch process.

**Figure 6:**
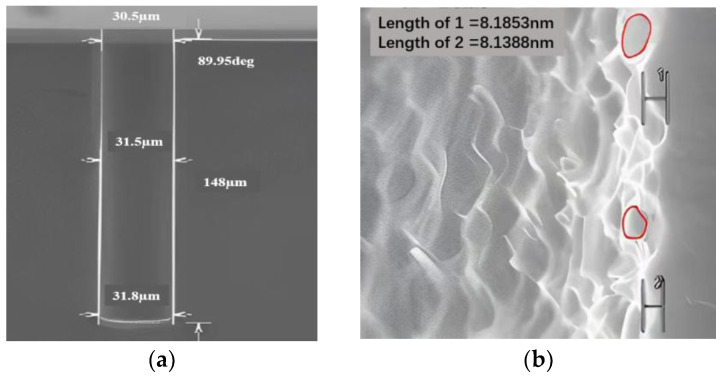
SEM morphology of the 30 µm hole. (**a**) Cross-section of the TSV hole prepared by the Bosch process; (**b**) local detail of the TSV hole (Reprinted with permission from Ref. [10], 2021, Wu, D.-W.).

At present, there are two etching methods for TSV: deep reactive ion etching (DRIE) and laser etching (LE) [25]. Deep reactive ion etching, also called “Bosch” etching, was first proposed by Bosch. As shown in process (1) in Figure 4, the Bosch process improves the anisotropy of TSV by alternately performing etching and protection steps to ensure the verticality of TSV vias, thus forming silicon vias. The flow chart of the etching process is shown in Figure 5. In each etching/passivation cycle of the Bosch process for TSV vias, SF_6_ is used to isotropically etch the silicon wafer, and the reaction process is shown in Formulas (1) and (2). C_4_F_8_ is used to deposit a protective layer on the inner wall of the TSV so that SF_6_ acts on the protective layer to prevent the silicon wafer from being excessively corroded. The reaction process is shown in Formulas (3) and (4). As the protective layer is eroded, the exposed silicon reacts with SF_6_ again, leading to repeated etching and protection until the etched size of TSV meets the established requirements [26]. Figure 6a is the cross-sectional view of the 30 μm diameter TSV after etching. It can be seen that the vertical orientation of the TSV holes prepared by the Bosch process is excellent, and the difference between hole’s bottom size and orifice size is small. However, alternating etching reaction and protection reaction will produce the scallop pattern on the wall of the TSV hole. The scanning electron microscope (SEM) image of scallop pattern is shown in Figure 6b, in which the red area is scallop pattern.

(1) $$SF_{6}+e^{-}\to {SF_{5}}^{+}+F+2e^{-}$$

(2) $$Si_{s}+4F_{g}\to SiF_{4g}$$

(3) $$C_{4}F_{8}+e^{-}\to C_{3}F_{6}+CF^{2}+e^{-}$$

(4) $$nCF_{2}\to CF_{\left(n\right)}$$

Laser etching [25] uses the high temperature of some light fields of the laser beam to vaporize the silicon edge through continuous high-temperature irradiation. This technology does not need to add other materials as a mask and directly forms holes through high-temperature irradiation at both ends of the TSV. At the same time, the sidewall formed by this method is inclined, which is easy to fill with a barrier layer or central metal conductor. This method also has some disadvantages, such as the rapid melting of the silicon irradiated by the laser and the rapid recovery of the silicon once the laser equipment is removed. The recovered silicon can no longer be restored to its original shape, resulting in a rough inner edge of the TSV, on which it is not easy to continuously deposit the seed layer. When the sidewall roughness increases, it can lead to performance degradation in both the electrical and mechanical aspects. The rough sidewalls result in an increased effective surface area, leading to higher resistance. The presence of surface defects and uneven regions along the TSV path contributes to elevated resistance, which can cause signal transmission losses and increased power consumption. The gaps between the rough sidewalls can cause unwanted signal coupling, resulting in crosstalk between adjacent TSVs. This interference disrupts the normal operation of neighboring circuits. The heightened sidewall roughness can diminish the material’s strength, and the uneven sidewall surfaces promote stress concentration, leading to increased brittleness and susceptibility to fractures. Under stress loading, rough sidewalls create additional stress concentration points and initiate crack formation. This accumulation of fatigue damage decreases the TSV’s lifespan. Therefore, precise control of sidewall roughness is crucial during TSV fabrication to ensure optimal electrical and mechanical performance. Employing appropriate fabrication methods and optimizing process parameters can effectively minimize roughness, thereby enhancing the overall performance and reliability of TSVs.

Regarding the ability to form holes with a high aspect ratio, DRIE is famous for its excellent ability to prepare holes. It can achieve an aspect ratio greater than 20:1, which is very important for manufacturing deep and narrow through holes. The anisotropic etching process of DRIE enables it to effectively etch a longitudinal depth while maintaining a small lateral width. Laser etching also has the potential to prepare holes with a high aspect ratio, but its aspect ratio is generally lower than DRIE. The beam diameter and energy distribution during laser etching may make it difficult to achieve a very high aspect ratio.

Regarding manufacturing efficiency and cost, DRIE is a relatively time-consuming process involving many steps, including deposition, lithography and etching; the stacking of these steps may lead to a long processing time. However, DRIE is a mature technology widely used in the semiconductor industry, which benefits from economic benefits in mass production and can reduce unit costs. Compared with DRIE, laser etching is usually a faster process because it directly uses lasers for denudation; the process is simplified and the manufacturing efficiency can be improved. However, the cost of laser equipment and maintenance may be high, which may have an impact on the overall cost.

Therefore, the precision of the processed TSV products is small and the error is large; thus, the deep silicon etching of TSV often adopts DRIE etching technology at present [27].

#### 2.1.2. Insulating Layer Deposition Process

**Figure 7:**
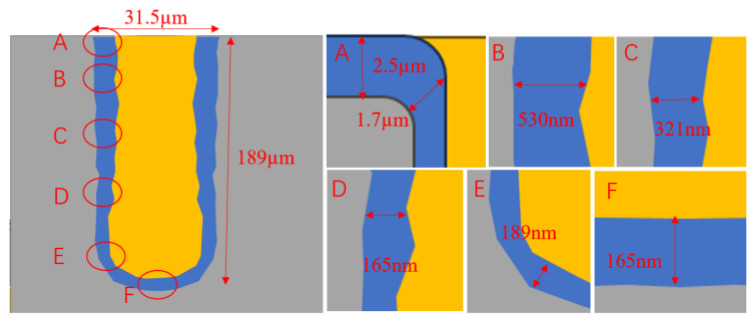
Cross-sectional view of SiO_2_ dielectric deposition in the TSV deep hole (Reprinted with permission from Ref. [10], 2021, Wu, D.-W.).

The insulating layer in the TSV hole is used to insulate the silicon substrate from the transmission channel in the hole, which effectively reduces the signal distortion and crosstalk caused by the signal leaking from the conductive metal to the silicon substrate. The quality of the sidewall isolation layer has become a main concern. The failure of the insulating layer may lead to leakage or other reliability problems, which directly affect the yield of silicon-based devices [28]. The methods of dielectric layer deposition mainly include high-temperature thermal oxidation, physical vapor deposition (PVD) and chemical vapor deposition (CVD), among which CVD includes plasma enhanced chemical vapor deposition (PECVD) and sub-atmospheric pressure chemical vapor deposition (SACVD) [29]. These two vapor deposition methods can meet the requirements of efficiency and shaping coverage when depositing silicon dioxide. Figure 7 shows the SEM cross-sectional view of SiO_2_ dielectric layer prepared in the TSV hole. In the figure, A to F are the deposition effects at different positions of the hole. It can be seen that the thickness of the SiO_2_ dielectric layer at the top of the TSV hole is relatively large. It gradually decreases after depositing the SiO_2_ film along the inner wall of the hole to the lower wall of the hole. Furthermore, the hole is completely covered with SiO_2_ dielectric material, and there is no missing area of SiO_2_ [30].

#### 2.1.3. Metal Deposition of Barrier Layer and Seed Layer

**Figure 8:**
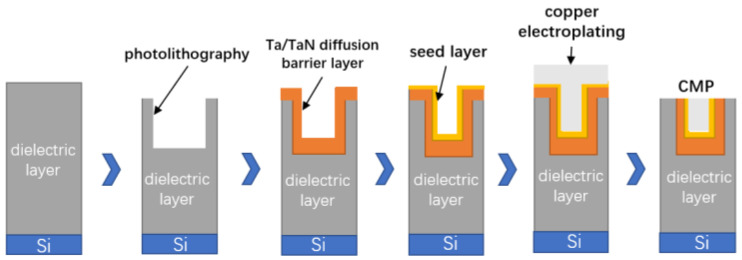
Schematic demonstration of the Cu.

Cu is generally used as the internal metal interconnection material of TSV vias in the manufacturing process of TSV. However, the application of copper in the interconnection lines faces some challenges. The diffusion speed of copper in the silicon dioxide medium is very fast, which easily leads to the serious degradation of the dielectric properties of the insulating layer and even leads to the deterioration of the device at low temperatures. Therefore, before electroplating Cu to fill the TSV vias, the barrier layer can be prepared to prevent copper from migrating to the silicon substrate, so that copper can be used in integrated circuits. Usually, cobalt, silicon tantalum nitride, titanium, titanium nitride, silicon nitride and tungsten nitride are used as barrier layers. Among them, titanium (Ti) is used as a seed layer and barrier layer because of its low resistivity and high thermal stability. Titanium is considered an excellent barrier material that can prevent copper from infiltrating at an annealing temperature of 400 °C and provides good adhesion between the copper and dielectric [31]. For the manufacture of high aspect ratio vias, it is difficult for ordinary deposition methods to deposit continuous copper on the sidewalls of the TSV. Ion Magnetron sputtering (IMP) or metal–organic chemical vapor deposition (MOCVD) methods are usually used to generate barrier layers and seed layers [32]. Figure 8 depicts the circulation of copper during the process of via hole technology procedure. Copper is deposited from the dielectric layer on the silicon wafer, and the dielectric film is patterned by photolithography. Then a thin film of Ta/TaN diffusion barrier layer is deposited on top of the dielectric pattern by PVD or CVD. It is typically necessary to deposit a copper seed layer beforehand for copper electroplating due to the low conductivity and poor nucleation behavior of Cu on the Ta/TaN layer, as well as the poor ductility of these barrier materials in previous studies [33].

#### 2.1.4. Center Conductor Filling

**Figure 9:**
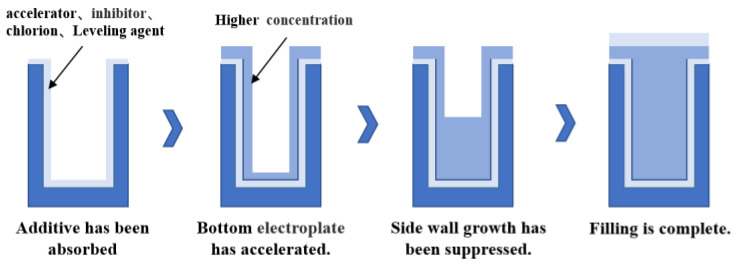
“Bottom-up” deposition process.

At present, the main metal materials filled with the central conductor of the silicon through hole are copper and tungsten. Because of its excellent conductivity, copper can be well integrated with the current process, and electroplating is generally used to fill copper [34]. Tungsten is also one of the most commonly used filling materials as a conductive material for TSV and is also very compatible with the current process. Tungsten has unique advantages that copper does not have. On the one hand, tungsten is a non-diffusion metal, and there is no need to deposit a barrier layer/seed layer, which reduces the manufacturing cost of TSV and shortens the manufacturing process. On the other hand, because the thermal expansion coefficient of metal tungsten is small, the gap with other materials is small. Hence, the thermal–mechanical stress of tungsten–silicon vias of the same size is much smaller than that of copper–silicon vias. Of course, tungsten also has some disadvantages, such as a higher resistivity than copper, which makes tungsten TSV not suitable for high-power signal transmission lines and power/ground wires. In addition, aluminum and other metals are used as conductor-filling materials. To sum up, which material to choose for filling depends on the purpose of the manufactured silicon via [35]. At present, the copper sulfate process system is mainly used for filling copper in TSV electroplating. Six elements make up the majority of the electroplating solution: sulfuric acid, copper ions, chloride ions, accelerators, inhibitors, and levelers. As shown in Figure 9, the ideal filling process is a bottom-up deposition process, which requires a reasonable proportion of different additives, such as inhibitors and accelerators, in the copper plating solution. This process can achieve the effect of “accelerating in the hole and inhibiting the hole”; this results in a silicon through hole structure with low resistivity, no cavity and high reliability [36].

**Figure 10:**
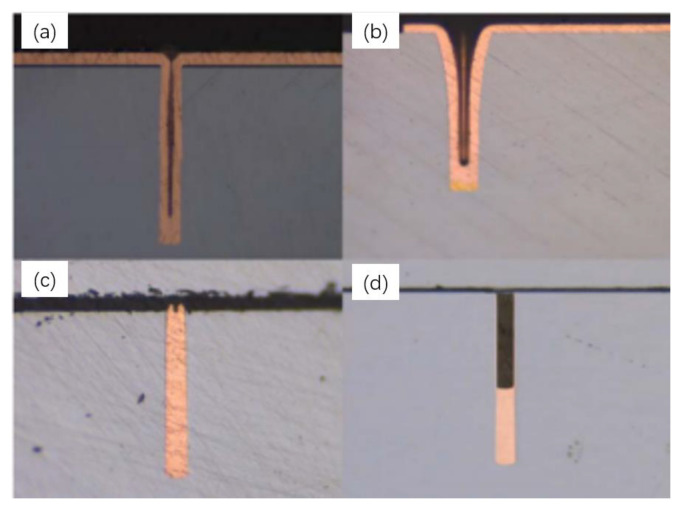
The filling morphology of the TSV electroplating with different processes; (**a**) not conformal; (**b**) conformal; (**c**) superconformal; (**d**) bottom-up (Reprinted with permission from Ref. [10], 2021, Wu, D.-W.).

**Figure 11:**
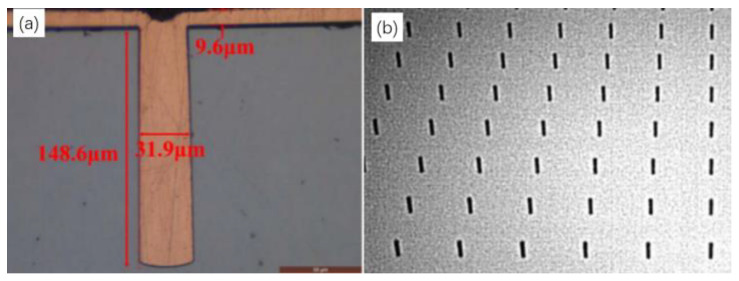
The morphology of TSV deep hole electroplating; (**a**) sectional view of TSV; (**b**) X-ray diagram of TSV after electroplating (Reprinted with permission from Ref. [10], 2021, Wu, D.-W.).

There are four kinds of filling processes for TSV electroplating, namely, conformal, not conformal, superconformal and bottom-up growth. The filling effects of different electroplating processes are shown in Figure 10. Figure 11a shows the SEM image of the Cu cross-section filled by deep-hole electroplating of a 30 μm TSV [10]. It can be seen that the bottom-up electroplating method can effectively realize the copper electroplating filling in TSV holes. Figure 11b shows the X-ray test results after TSV electroplating. It can be seen that the top-down electroplating process had good stability and uniformity. The requirements for mass manufacturing were met as every hole was entirely plated, and there were no missing plating phenomena.

#### 2.1.5. Wafer Front CMP Process

**Figure 12:**
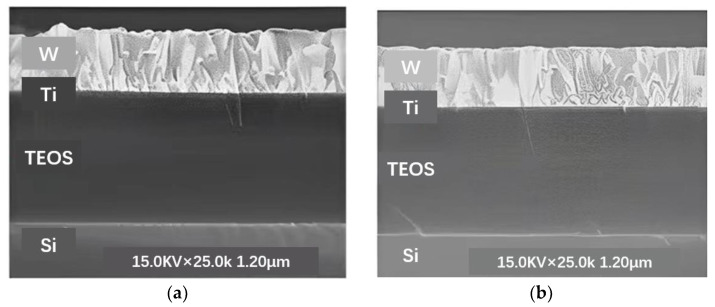
Comparative cross-section of the chip with or without the CMP process; (**a**) before CMP SEM image; (**b**) the chip has a CMP cross-section (Reprinted with permission from Ref. [10], 2021, Wu, D.-W).

Chemical mechanical planarization (CMP) is a wet polishing technology that combines chemical force and mechanical force to ensure the stability and reliability of high-density devices [37]. Figure 12 shows a cross-sectional view of the multilayer interconnection process without the CMP process and with the CMP process [38]. CMP technology is introduced in the TSV process to remove the SiO_2_ dielectric layer, barrier layer and seed layer on the silicon surface [39]. Excess copper produced by electroplating is removed from the surface by the CMP process, and a rapid copper removal rate and high consistency are required. Secondly, in order to remove the barrier layer, the polishing solution must have speed selectivity and minimize defects. Finally, polishing must be carried out on the oxide layer. Therefore, the most important thing in the metal–CMP process is to minimize the polishing selectivity between the metal interconnect materials and barrier materials, as well as the SiO_2_. In the CMP process, when the oxide on the copper surface is removed, the chemical composition of the polishing solution will oxidize the newly exposed metal surface, which is then mechanically ground until all of the extra copper metal is gone.

Gaps and partial filling are common defects encountered in the process of TSV (through-silicon Via) filling. These issues present challenges to the reliability and performance of TSVs during the packaging process. To address these problems, researchers have been continuously improving and developing new filling techniques. Here, we highlight some of the latest advancements in filling technology. Electrochemical deposition is a method that uses electrochemical reactions to deposit metal fillers into TSVs. By controlling parameters such as current density and additives, uniform filling can be achieved, reducing gaps and partial filling issues. Optimizing material selection, current density distribution and additive formulations can improve filling effectiveness. Magnetron sputtering is a method that utilizes a magnetic field to guide the deposition of metal ions. By optimizing sputtering process parameters and material selection, a more uniform filling can be achieved, reducing gaps and partial filling. Additionally, post-processing techniques such as multilayer filling and annealing can improve filling quality. High-speed filling techniques aim to improve filling effectiveness by altering filling speed and fluid dynamics conditions. Optimizing the rheological properties of the filling media, controlling filling speed, and improving fluid dynamics can reduce gaps and partial filling while enhancing filling quality and efficiency. These latest filling technologies are currently being explored to overcome the common issues of gaps and partial filling encountered during the TSV processes. However, it is important to note that these technologies are still undergoing refinement and development, and their applicability may be influenced by specific application requirements and process conditions. Therefore, the selection of an appropriate filling technique should be based on careful consideration of the specific circumstances and desired outcomes.

#### 2.1.6. Wafer TSV Back Outcrop Process

**Figure 13:**
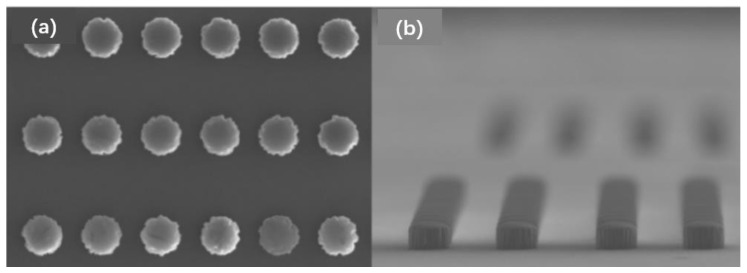
The morphology of the copper column after back outcrop; (**a**) top view of the Cu column; (**b**) side view of the Cu column (Reprinted with permission from Ref. [10], 2021, Wu, D.-W).

The core technology for 2.5D TSV transfer substrates, which also includes dry/wet etching and wafer thinning, is TSV back outcrop technology. As shown by processes (10) and (11) in Figure 4, the wafer thinning process is an important process that needs great attention. In the TSV interposer process, the wafer thickness needs to be increasingly smaller. Because thicker wafers are difficult to meet the heat dissipation and packaging requirements of high-end chips, thin wafers are often required to be as thin as possible in the semiconductor field. Moreover, the smaller the TSV depth, the corresponding interconnection delay and loss will also be reduced. After the thinning of the TSV transfer substrate, it is generally necessary to expose the TSV copper column from the back by a dry or wet etching process so as to realize the electrical signal connection on the back of the subsequent wafer [40]. Generally, there are two methods: dry process and wet process. The wet process is a pure chemical etching method, whereas the dry process is an etching process combining physical bombardment with chemistry. The Cu outcrop process is related to the success of the electrical signal connection on the back of the subsequent wafer, which requires a high uniformity of exposure. Considering that the selection ratio of silicon-to-silicon dioxide needs to reach 70:1, the dry process was chosen. The results of dry copper exposure on the back of TSV adapter board are shown in Figure 13.

#### 2.1.7. Temporary Bonding/De-Binding Process (TBDB) for Ultra-Thin Wafers

**Figure 14:**
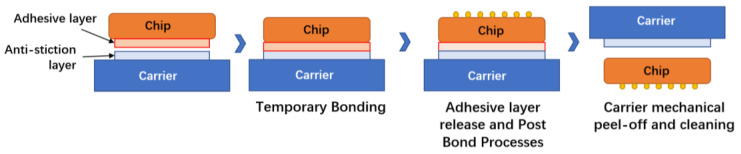
The TBDB process flow.

TBDB is one key technology in enabling the 2.5D/3D integration of semiconductor devices [41]. Temporary bonding/debonding (TBDB) technology has been put forward in the semiconductor industry to avoid fragmentation after wafer thinning. In this technology, the wafer is temporarily bonded to a hard slide to provide sufficient mechanical support. After the processing and interconnection are completed, the TSV silicon transfer substrate is peeled off from the temporary slide without loss [42]. The process flow of the TBDB is shown in Figure 14.

### 2.2. Development Status of TSV Technology at Home and Abroad

In the past decades, many countries’ enterprises and institutions have conducted extensive research on silicon vias; these are mainly INTEL and IBM in the United States, ASET in Japan, SAMSUNG in South Korea, etc., which have conducted in-depth research on the process and structure. They have all achieved good research results, thus laying a technical foundation for the large-scale application of TSV.

#### 2.2.1. Domestic Development Status of TSV

(1) Homogeneous high-density 3D integration has made a comprehensive breakthrough and is being industrialized.

In China, the Ministry of Science and Technology, the former General Armament Department, the Science and Technology Bureau and other ministries and commissions have always advocated for promoting the informatization and miniaturization of weapon systems, and arranged high-density TSV-related technical research plans at all levels of systems, modules and chips. In their pre-research and funding plans, numerous domestic universities and research institutions have set up targeted technical research in the areas for microelectronics, optoelectronics, micro-nano electronics, electronic components and advanced manufacturing technologies [43]. Domestic TSV stereoscopic integration technology research institutes have completely overcome high-density stereoscopic integration technology and created matching samples after investigating TSV’s associated technologies.

China’s Xi’an Institute of Microelectronics Technology developed the first 3D integrated radiation-reinforced tiled 4 Mb static random-access memory (SRAM), four-layer stacked 4 Mb SRAM and 8 Mb SRAM samples based on silicon adapter plates, in 2014, 2015 and 2016 respectively, all on the basis of the military standard production line. In the stacked samples, the area of TSV adapter plates is about 20 mm × 20 mm, the thickness is 250 μm, the diameter of TSV through holes is 100 μm and the ratio of depth to width is about 2.5:1. In the SRAM chip, the diameter of TSV via is 30 μm, the depth is 130 μm, and the ratio of depth to width is about 4:1. This indicates that China has mastered the ability of high-density integration based on 4:1 TSV via, 250 μm silicon interposer and interposer with no less than two layers on one side, and has also laid a technical foundation for the research of super-large TSV interposer technology for 3D heterogeneous integration. With the support of the national project, Xi’an Institute of Microelectronics Technology has built a platform based on 3D integrated memory in order to surpass the international advanced 3D integrated memory [44,45].

(2) The development status of heterogeneous 3D integration based on a silicon interposer.

**Figure 15:**
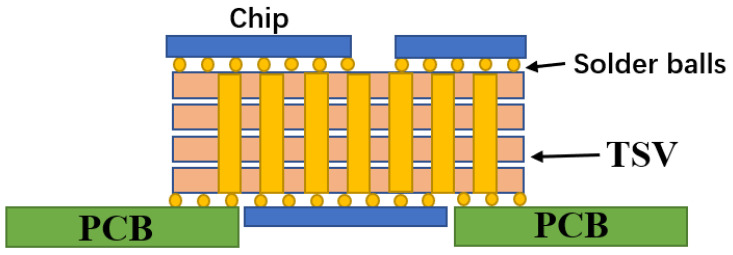
ITRI’s TSV adapter board structure.

China’s research on heterogeneous 3D integrated manufacturing technology is mainly concentrated in Taiwan Province, which is already at the leading level in the world. The Taiwan Province Industrial Technology Research Institute (ITRI) established the 3D integration alliance—Ad-STAC. In 2013, ITRI successfully completed the double-sided interconnection between the TSV adapter board and different functional chips, as shown in Figure 15 [46,47]. In 2014, Taiwan Semiconductor Manufacturing Company (TSMC) successfully realized the technology related to the micro-assembly of logic chips and interposer wafers.

Although mainland China has accumulated some experience in converting narrow-pitch to wide-pitch adapters for homogeneous high-density integration, the research on the bonding assembly process of heterogeneous integration chips with different functions and materials, the influence of assembly material selection, the process sequence and key parameters on the performance of 3D heterogeneous integration modules are still in their infancy.

#### 2.2.2. Development Status of TSV Abroad

Wafer foundries such as TSMC, United Microelectronics (UMC) and Global Foundries have promoted the development of TSV heterogeneous stacking technology by virtue of their own advantages in developing and producing intermediate layers. With its “Foveros” technology, Intel is the only integrated device manufacturer (IDM) trying to compete in this field. Regarding 3D stacked storage, the competition is mainly concentrated among the IDM “Big Three” Samsung, SK Hynix and Micron, and these three companies will continue to dominate the stacked storage market.

**Figure 16:**
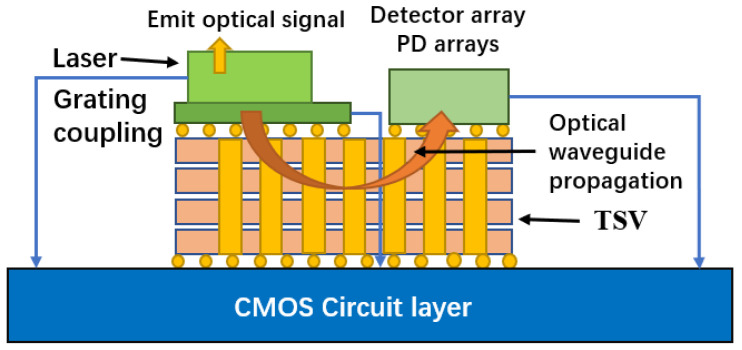
Hybrid optoelectronic integration based on TSV.

TSV is very important for applications such as complementary metal oxide semiconductor (CMOS) image sensors (CIS), high bandwidth memory (HBMS) and switchboards. Because the surface of the CIS chip is photosensitive, the electrical signal of the CIS chip must be output from the back; TSV now serves as the fundamental CIS chip electrical connection structure. Three-dimensional integration technology based on TSV technology, which is engaged in by the Massachusetts Institute of Technology and Eindhoven University of Technology in the Netherlands, is the best way to realize a photoelectric integrated micro-system. Figure 16 shows a schematic diagram of a photoelectric micro-system [48].

**Figure 17:**
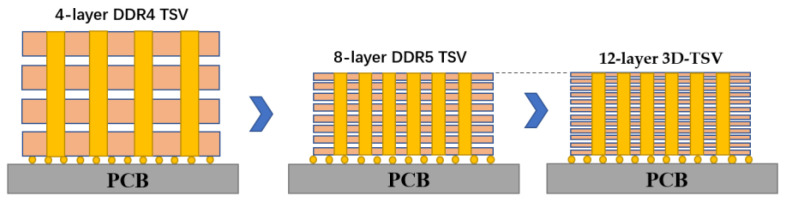
The 3D-TSV chip packaging process.

At present, TSV technology is the preferred connection method for high-end storage. The most successful manufacturer is Samsung. Samsung unveiled the first 64 GB DDR4 memory module in the world with TSV 3D packaging technology. To increase capacity and performance, DDR4 employs Samsung’s cutting-edge 20 nm and 3D TSV via-middle packaging technology, which connects the chips or silicon wafers vertically [49]. At the HotChips 33 (2021) Conference, Samsung introduced the DDR5 memory with an eight-layer TSV package that they are developing. Compared with the four-layer TSV carried by DDR4 of Samsung Electronics, its memory capacity will be twice that of DDR4, and a single DDR5 with 512 GB will be realized in the future. Through package optimization, Samsung decreased the chip pitch by 40% and employed thin wafer technology, resulting in a DDR5 height of an eight-layer TSV package that was lower than that of a four-layer DDR4 package and had better heat dissipation capacity [50]. In addition, Samsung Electronics has successfully developed a new process for packaging twelve-layer 3D TSV chips. This is the first process in the industry to use a twelve-layer TSV 3D package, compared with only eight layers before. The shell thickness of the new twelve-layer 3D TSV chip is 720 μm, which is equivalent to the current eight-layer HBM2. Maintaining the same thickness allows customers to use new twelve-layer packaging products without changing the existing design, which means that products with a larger capacity can be directly used. As shown in Figure 17, HBM is a memory chip based on a multilayer stack. Today, HBM can produce twelve-layer stacks, and it is estimated that it will produce more than sixteen-layer stacks in the near future. Of course, if there is no TSV connection, this is impossible [51].

## 3. Optimization and Reliability Analysis of TSV Technology

This review analyzes and studies the three key processes of TSV hole etching, deep hole electroplating filling and wafer CMP in order to better understand its implementation and related challenges in the industry. By analyzing the improvement measures and integrating the process flow, the reliability of the TSV process can be improved.

### 3.1. TSV Technology Optimization

#### 3.1.1. Optimization of the Etching Process

**Figure 18:**
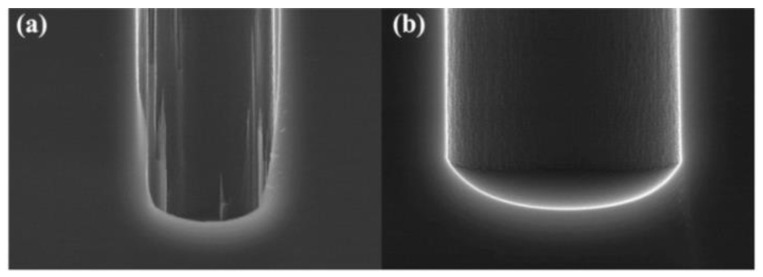
Comparison diagram of micro grass at the bottom of the TSV hole before and after optimization; (**a**) there are micro sketches after etching; (**b**) no micro-sketch after etching (Reprinted with permission from Ref. [10], 2021, Wu, D.-W.).

The local active etching groups in the hole eventually become fatigued as the depth–width ratio rises throughout the etching process. This causes a number of secondary issues to arise, including reactive-ion etching (RIE) lag, nicking, micro-grass, etching halt and loss of critical dimension (CD) control. In order to optimize the etching sidewall morphology, Daowei Wu [10] proposed a preparation method of a photoresist AZ6130/SiO_2_ composite structure mask, and the optimal selection ratio of a composite mask as 2.3. On this basis, the etching process parameters of the TSV holes are optimized. By comprehensively shortening the etching time, prolonging the passivation time and increasing the bias power to 90~120 W, the problem of micro-grass at the bottom is successfully solved, as shown in the SEM images before and after optimization in Figure 18.

**Figure 19:**
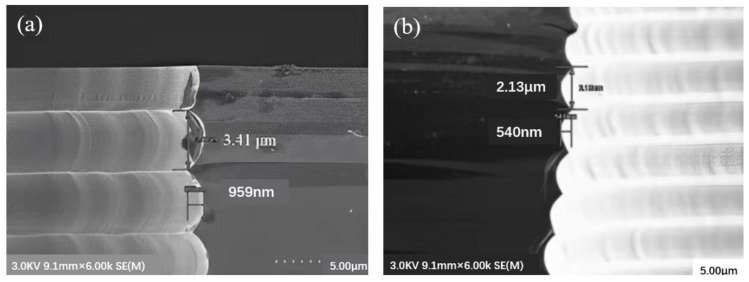
Scallops on the top side wall of the hole, with an opening of 80 μm (**a**) before optimizing DRIE parameters, the maximum width and depth of scallop were 3.41 μm and 959 nm, respectively; (**b**) the optimized DRIE parameters: the maximum width and depth of scallop are 2.13 μm and 540 nm, respectively (Reprinted with permission from Ref. [52], 2022, Feng, Liuhaodong).

**Figure 20:**
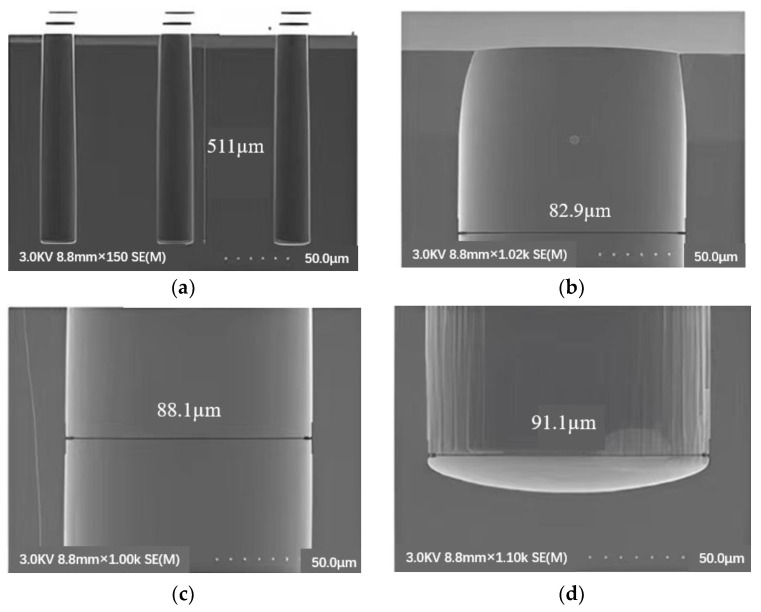
Profile after the formation and removal of the thermal oxide layer on the shaft wall. (**a**) Full-hole profile; (**b**) top profile; (**c**) the middle section; (**d**) bottom profile (Reprinted with permission from Ref. [52], 2022, Feng, Liuhaodong).

Liuhaodong Feng et al. [52] used a combination of blind etching and back thinning instead of penetrating etching. By optimizing the deep DRIE formula and then smoothing the oxide layer by combining thermal oxidation and wet etching, the sidewall of the hole becomes very smooth: the largest scallop size is reduced to 2.13 µm in width, 540 nm in depth and 43.7% in depth, as shown in Figure 19. In DRIE, the through hole is often formed at the bottom opening of the through hole through the combination of blind hole DRIE and back grinding process, which completely avoids the notch structure. Figure 20 shows the outline of the hole produced by the smoothing of the side wall.

**Figure 21:**
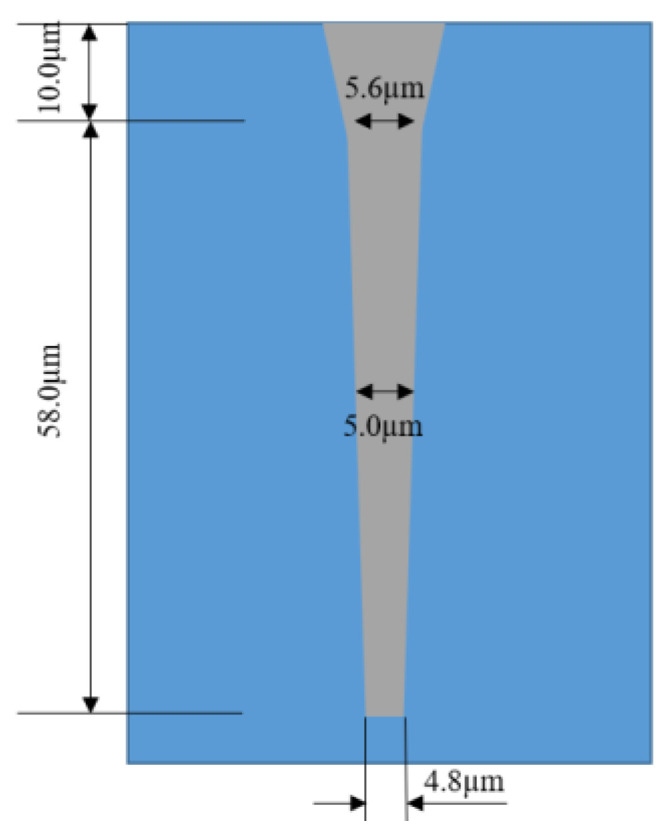
The model of the TSV etching morphology with the SiO_2_ about 10 μm thick on the surface of the Si substrate.

Park et al. [53] optimized the RIE conditions of 100 W RF power and 30 SCCM (standard cubic centimeter/minute) for 30 min, which reduced the average depth of the scallops from 230 nm to 20 nm, resulting in a smooth profile of the sidewall groove. Frasca et al. [54] reported that using 40% potassium hydroxide at 60 °C to form a Michelangelo step can prevent the formation of scallops, and conformal copper filling can control the critical aspect ratio phenomenon in DRIE by a micro-loading effect and RIE hysteresis. Gerlt et al. [55] realized the reduction of RIE lag by adjusting the two-step process parameters of Bosch. In the case of simplicity and complete adaptability, when the etching depth is 50 µm, they reduce the RIE lag to below 1.5%. Hong Zhao et al. [56] deeply etched SiO_2_ micropores with an etching rate of 0.612 µm/min, an etching selectivity of 2.122 and an etching angle of 80.573. Thereby, the TSV with small aperture and high aspect ratio is optimized to eliminate the “undercut” effect of aperture and meet the requirements of preparing the aperture profile with a small aperture and high aspect ratio on the active chip. The etching morphology is shown in Figure 21.

#### 3.1.2. Optimization of Electroplating Filling Technology

**Figure 22:**
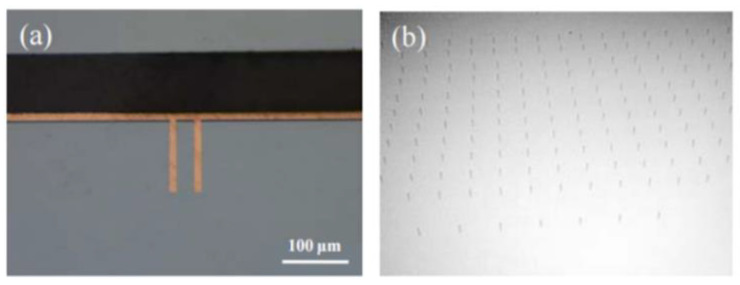
The effect diagram after electroplating and filling; (**a**) grinding plate diagram after electroplating; (**b**) X-ray diagram after electroplating (Reprinted with permission from Ref. [10], 2021, Wu, D.-W.).

In the process of electroplating copper filling, defects such as voids or cavities will lead to serious reliability problems, so it is very important to optimize the process of electroplating filling to achieve a good filling effect for functional reliability. Based on this, Daowei Wu [10] suggested that the electroplating should adopt a segmented current mode to increase the jitter setting. The process parameters of segmented electroplating obtained from the test are shown in Table 1, and the best effect was obtained when the jitter frequency was 20 Hz. Figure 22 shows the effect diagram after electroplating filling with the parameters in Table 1.

Yuzhe Wang et al. [12] studied the bottom-up electroplating process and obtained the TSV processing technology with a silicon wafer thickness of 370 μm and a via hole diameter of 60 μm.

**Figure 23:**
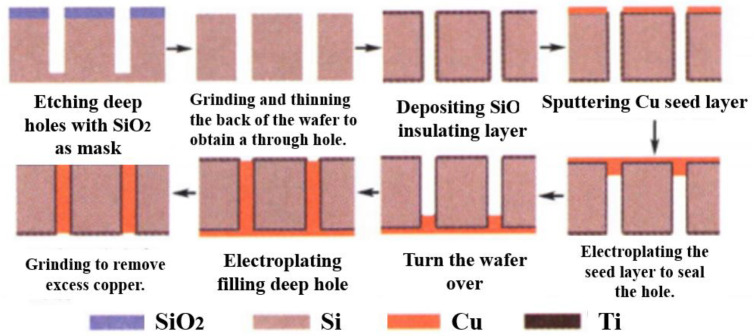
The processing flow of the step-by-step filling of the TSV on both sides of the through hole (Reprinted with permission from Ref. [57], 2022, Tian, M.).

**Figure 24:**
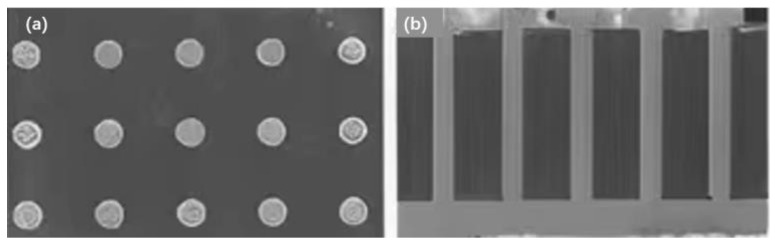
SEM images of transverse and longitudinal sections of the fully filled 30 μm × 300 μm TSV; (**a**) SEM diagram of the cross-section; (**b**) SEM diagram of the longitudinal section (Reprinted with permission from Ref. [57], 2022, Tian, M.).

Miao Tian et al. [57] adopted a two-step filling electroplating method, and the process flow is shown in Figure 23. First, electroplating was performed at a low current of 0.5 mA/cm^2^ for 10 min, and then electroplating was performed at a current of 5 mA/cm^2^ for 5 h. By observing the transverse and longitudinal sections of the TSV, it can be confirmed that copper completely filled the whole via hole, and no slit error or bump/void defect was observed. The pore-free filling of the TSV with a pore size of 30 μm, a hole depth of 300 μm and a depth–width ratio of 10:1 was realized. Figure 24 shows the filling effect.

#### 3.1.3. CMP Technology Optimization

Kang Zhang et al. [58] provided an optimized TSV CMP matching process, and the process parameters are shown in Table 2, which meet the requirements of wafer surface planarization in TSV technology.

In order to ensure the polishing effect of the barrier layer, it is necessary to improve the removal rate selection ratio of various materials in the barrier layer by controlling the composition of the polishing solution, to finally achieve global planarization of the wafer.

**Figure 25:**
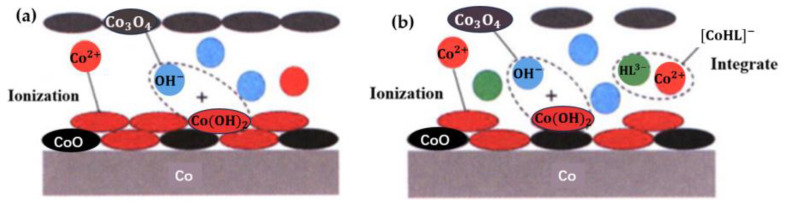
The complexation mechanism between Co and HEDP; (**a**) polishing solution without HEDP; (**b**) polishing solution containing HEDP (Reprinted with permission from Ref. [61], 2021, Hu, Lianjun).

Xuyang Liu [59] found that the glycine–H_2_O_2_ system was more beneficial in improving the electrochemical corrosion and removal rate of Cu, and corrosion inhibitor 1,2,4-triazole (TAZ) and surfactant betaine were introduced into the system to improve the surface quality of the copper wafer and reduce the surface roughness. It was also studied that available guanidine hydrochloride and hydrogen peroxide can effectively improve the removal rate of Ta and promote the corrosion of Ta. In 2020, O. Kwon et al. [60] studied the effect of a complexing agent on the oxidation/dissolution of Co in an alkaline polishing solution. Glycine, ethylenediamine tetraacetic acid (EDTA) and citric acid were tested, respectively. The results show that the presence or absence of H_2_O_2_ affects the co-solubility rate of the complexing agent. In the absence of H_2_O_2_, the co-dissolution rate of glycine was higher than that of EDTA and citric acid, while the dissolution rate of EDTA was the highest in the presence of H_2_O_2_. In 2021, L. J. Hu et al. [61] reported that hydroxyethylidene diphosphonic acid (HEDP) could be used as a complexing agent for CMP of a Co-based barrier layer of multilayer Cu interconnection. Figure 25 shows the complexation mechanism between Co and HEDP.

**Figure 26:**
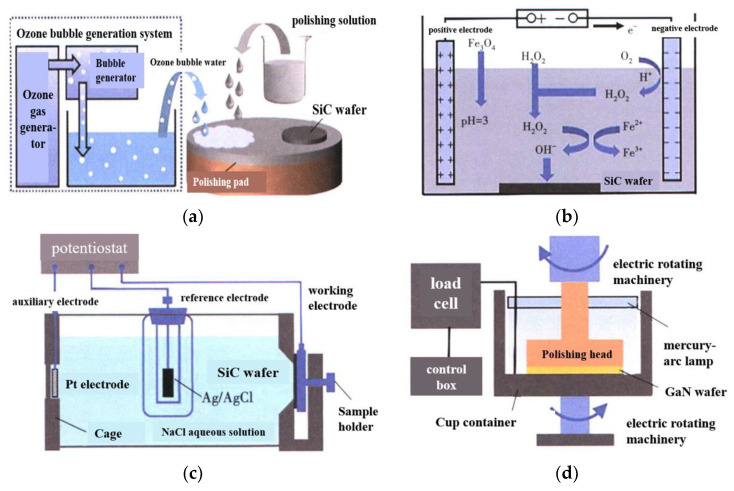
The new technology for improving the CMP effect; (**a**) The CMP strengthening effect of a polishing solution with ozone bubbles on the SiC substrate; (**b**) schematic diagram of a polishing SiC single crystal after the electro-Fenton reaction; (**c**) schematic diagram of the anodic oxidation of NaCl aqueous solution; (**d**) schematic diagram of the photochemical enhanced CMP (Reprinted with permission from Refs. [62,63,64,65]).

Figure 26 shows some new technologies that can improve the CMP effect. M. Uneda and K. Fujiil [62] studied the enhancement of the polishing effect of a polishing solution using ozone bubbles, as shown in Figure 26a. J. Y. Deng et al. [63] proposed that the electro-Fenton reaction improved the activity of hydroxyl groups in the polishing solution, as shown in Figure 26b. X.Z. Yang et al. [64] put forward the method of anodic oxidation in an electrochemical process, which effectively improved the surface quality of the SiC wafer, as shown in Figure 26c. L. W. Ou et al. [65] used ultraviolet light to irradiate a GaN wafer, which excited GaN to generate electron–hole pairs, and the synergistic effect of oxidant effectively improved the removal rate of GaN material, as shown in Figure 26d.

### 3.2. TSV Reliability Analysis

With the development of 3D integrated packaging technology, TSV technology has become one of the most critical technologies in 3D stacked packaging. As an important physical and electrical connection between chips, the reliability of TSV is undoubtedly the key to determining the reliability of 3D integrated devices.

#### 3.2.1. Thermal Stress Analysis

Three-dimensional-integrated devices are affected by thermal, electrical and other stresses generated in TSV process, which significantly damages the reliability of 3D devices. In the TSV structure, the thermal expansion coefficient of silicon is about 2.5 × 10^−6^/°C, and that of copper is about 17.5 × 10^−6^/°C. Due to the significant difference in the thermal expansion coefficient between silicon and copper, when the process temperature changes, significant thermal stress will occur. These thermo–mechanical stresses will directly lead to various reliability problems, such as internal cracking or interface degradation of TSV, which will lead to the failure of the signal transmission ability of TSV, which has a great impact on circuit performance. If the 3D integrated circuit industry can develop continuously, it will become increasingly important to control the power consumption and performance stability of the chip. Because of the complex structure and numerous devices of 3D integrated circuits based on silicon vias, research on the thermal–mechanical stability of the system has become very critical. Shuo Wang et al. [13] proposed an effective method to reduce thermal stress by shallow trench isolation (STI). STI stands for shallow trench etched between the TSV and active region, and its function is to release stress. The effects of the TSV diameter, depth–width ratio, distance and shape on thermal stress were analyzed and summarized. At the same time, it was concluded that the maximum stress of TSV structure increases with the increase of copper column diameter. With the increase of the thickness of the SiO_2_ layer, it decreases gradually; it gradually increases with the increase in height, and when the height exceeds a certain value, the stress value tends to be stable; with the increase in TSV spacing, it decreases gradually. If the spacing exceeds a certain value, the degree of stress reduction will slow down. Reducing the difference in thermal expansion coefficient between materials is also the most effective way to reduce the thermal stress level and improve the reliability of the TSV.

Min Zhang et al. [66] measured the residual stress of TSV–Cu/TIW/SiO_2_/Si interconnection structures under different thermal loads by the ion-beam layer removal (ILR) method and observed the failure mode of the interconnection interface. The influence mechanism of the microstructure evolution on the residual stress gradient was analyzed. By measuring the stress using the ILR method, it was concluded that during the thermal cycle, residual stress was generated and accumulated on the interconnection interface, which increased the KAM value and promoted the recrystallization and nucleation of the TSV–Cu grains. Therefore, many new grain boundaries were formed, which led to a decrease in grain size and an increase in tensile stress at the interface of the TSV–Cu/TiW.

**Figure 27:**
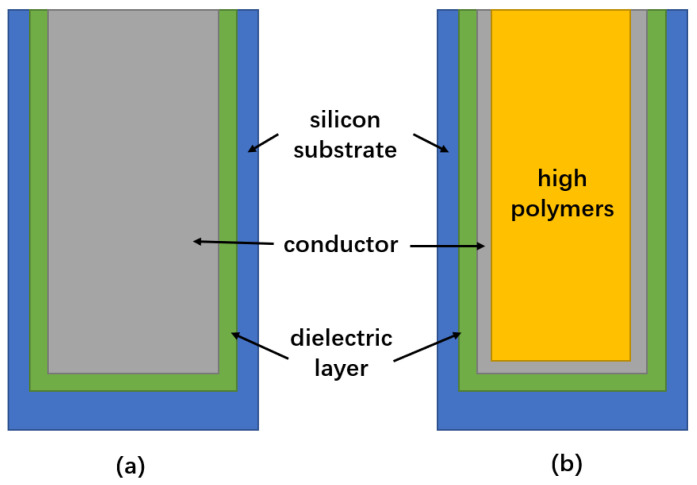
Silicon via shape type; (**a**) cylindrical silicon through hole; (**b**) annular silicon through hole.

Song Wei [24] filled TSV with various metals while researching the connection between thermal stress and copper, tungsten and aluminum. The variation of the TSV structural characteristics and the trend of thermal–mechanical stress were investigated, and it was determined that changes in TSV height, diameter and inclination angle impacted the thermal stress value. Finally, the relationship between different shapes of TSV and thermal stress was simulated. The cylindrical silicon via is a widely used and relatively simple structure among TSV structures, commonly employed for three-dimensional interconnections. It consists of three layers arranged from the inside out: a central conductor, an insulating layer (usually made of silicon dioxide) and a silicon substrate. In this configuration, the signal is transmitted through the central conductor. The annular through hole is a cylindrical structure as a whole. Another variation of this structure includes an organic polymer layer as the central filling material, followed by a metal layer, an insulating layer and a silicon substrate. Apart from the innermost layer, the composition of the remaining layers remains consistent, maintaining the cylindrical shape, as shown in Figure 27. It is concluded that the thermal–mechanical stress of the cylindrical TSV is the smallest and that of the annular TSV is the largest under the same parameter conditions, but the annular TSV is the least affected by the change of structural parameters, so the thermal–mechanical stability is better.

Jun Xie [67] obtained that the stress and strain of the TSV interconnection structure change with the temperature cycle through simulation analysis; its maximum stress and strain gradually increased after four thermal cycles. In addition, it was found that the micro-bump material had a significant influence on the stress of the TSV interconnect structure. Among the selected factors affecting the stress, the order of influencing factors was as follows: micro-bump material > SiO_2_ layer thickness > TSV spacing > micro-bump height > micro-bump diameter > copper column diameter > copper column height. By a single-factor analysis, the micro bump materials, the thickness of SiO_2_ layer and the TSV spacing with great influence factors were selected, and it was concluded that the stress and strain of the TSV interconnection structure were the largest and the smallest when the micro bump materials were Pb_90_Sn_10_ and Pb_37_Sn_63_.

#### 3.2.2. Thermoelectric Coupling Analysis

**Figure 28:**
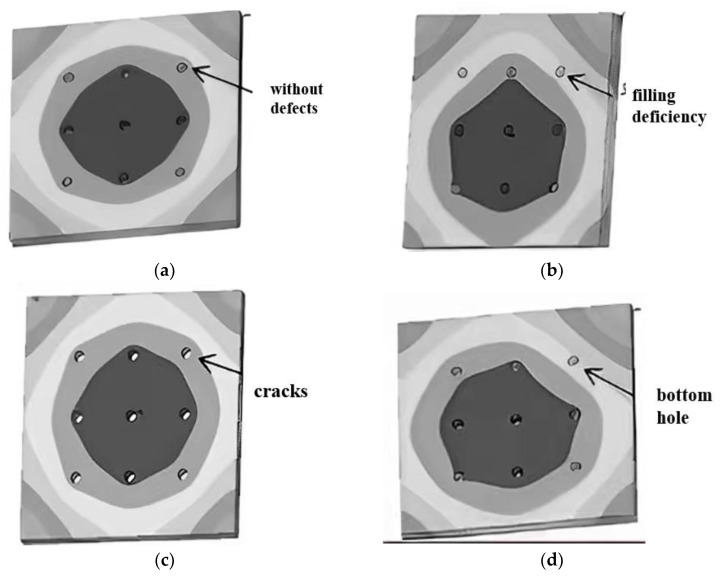
Nephograms of the temperature distribution of the TSV layer under various defects; (**a**) nephogram of the temperature distribution of the TSV layer without defects; (**b**) nephogram of the temperature distribution of the TSV layer under filling deficiency; (**c**) nephogram of the temperature distribution of the TSV layer under cracks; (**d**) nephogram of the temperature distribution of the TSV layer under the bottom hole (Reprinted with permission from Ref. [69], 2018, Nie, L.).

In the process of developing a TSV, it may be seriously hindered by several physical field coupling effects. The main reason is that the coupling process of multiple physical fields is very complicated, and the distribution of thermal field, electromagnetic field and structural field are interrelated and interactive. Therefore, the multi-physical field collaborative modeling and analysis of TSV is a core scientific problem to be solved. However, the above research focuses on one factor, and there is little research on the influence of the interaction of physical dimensions such as diameter and distance on reliability under the electro-thermal coupling effect. Therefore, Tao Zhang et al. [68] used the finite element simulation analysis method to study the influence of different diameters and pitches of TSV structure on the temperature distribution and stress distribution of thermoelectric coupling under the condition of electrothermal coupling. Lei Nie et al. [69] established the finite element model of 3D packaging of TSV under three typical defects and simulated and analyzed these by using the principle of thermoelectric coupling. According to the simulation results, different defects lead to different temperature distribution patterns, as shown in Figure 28. Therefore, monitoring the packaging temperature of TSV can detect and locate internal faults in TSVs.

#### 3.2.3. Thermal Torsional Vibration Coupling Analysis

With the wide use of TSV interconnection technology, the working environment of the TSV interconnection structure is more complicated, and it is often subjected to combined loads. In practical applications, the TSV interconnection structure in 3D integrated circuits will be affected by the periodic changes in the working environment temperature, warping deformation caused by thermal stress and torsion during the assembly and use of integrated circuits, which will seriously affect the reliability of the TSV interconnection structure. Vibration load is also an important environmental load that leads to the failure of electronic devices, and about 20% of electronic device failures are caused by vibration. Therefore, in order to further analyze the reliability of the TSV interconnection structure under a combined load, it is necessary to study the reliability of the TSV interconnection structure under a thermal–torsional–vibration combined load. Jun Xie [67] analyzed the reliability of the model under random vibration load and obtained the distribution of the stress and strain of the TSV interconnection structure with the magnitude of stress and strain as the reliability evaluation index. The fatigue life prediction and structural parameter optimization of TSV interconnection structure under thermal–torsional–vibration combined loading were carried out. The fatigue damage superposition method was used to predict and calculate the fatigue life of the TSV interconnection structure under thermal–torsional combined loading and random vibration loading, respectively, and the fatigue damage rates under two loading conditions were obtained. The fatigue life under thermal–torsional–vibration combined loading was obtained by linear superposition. Based on the corresponding surface and simulated annealing algorithm, the structural parameters of the TSV interconnection structure under combined thermal and torsional loading were optimized.

#### 3.2.4. Analysis of Electrical Characteristics

With the development of TSV technology, the aperture and spacing are getting smaller and smaller, and the corresponding electromigration reliability problem is more prominent. There are few international reports on the reliability of TSV power transfer. Pak, J. et al. [70] used the finite element method to predict the failure position of a single TSV electromigration. Frank et al. [71] studied the electromigration characteristics of a TSV adapter by finite element analysis and found that thermal–mechanical stress was the main factor affecting the electromigration of the TSV. They compared the temperature and Joule heat distribution of the TSV connection structure under different current densities and ambient temperatures. With the increase in temperature and current density, the Joule heat of the TSV increased, thus accelerating the failure of electromigration. Rui Ma et al. [72] studied the influence of TSV length and aperture on the temperature field distribution and electromigration of the TSV interconnection structure.

**Figure 29:**
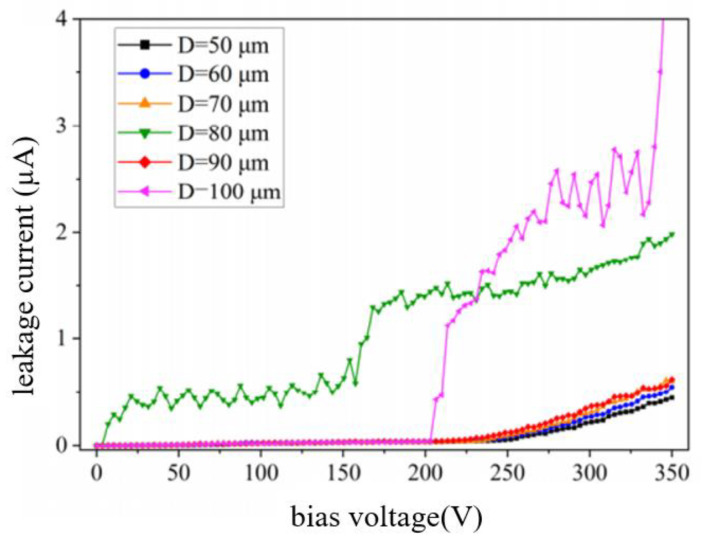
Leakage current test results of PECVD oxide film with a thickness of 500 nm on the substrate surface in silicon blind holes with a diameter of 50∼100 µm and a spacing of 60 μm (Reprinted with permission from Ref. [52], 2022, Feng, Liuhaodong).

**Figure 30:**
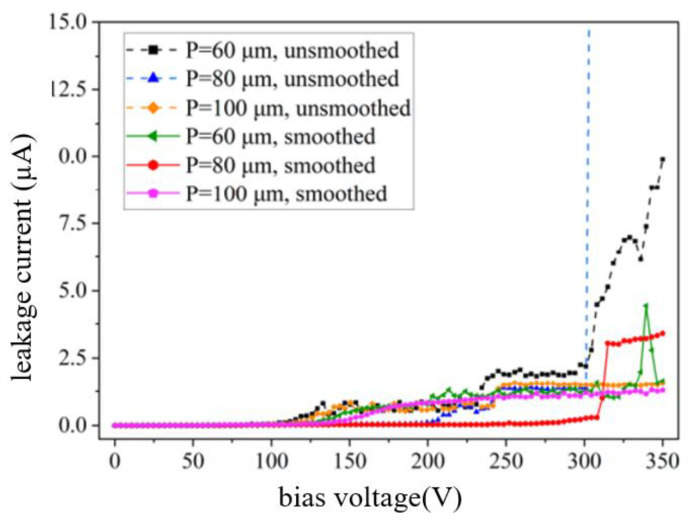
Leakage current test results (60 μm, 80 μm and 100 μm) of PECVD oxide film (substrate surface thickness is 500 nm) formed in silicon blind holes with a diameter of 80 μm, in which some hole sidewalls are smooth and some are not (Reprinted with permission from Ref. [52]. 2022, Feng, Liuhaodong).

Liuhaodong Feng et al. [52] studied the relationship between dielectric characteristics and the hole diameter of holes with non-smooth side walls. Figure 29 shows the dependence of 300 µm deep blind holes on dielectric characteristics without smooth side walls. The relationship between dielectric properties and hole spacing of holes with uneven and smooth side walls was also studied. Figure 30 shows the hole spacing dependence of dielectric properties of holes with uneven and smooth side walls.

From the existing literature and the above analysis, we know that the parasitic effect of silicon vias on 3D integrated circuits can not be ignored, especially since the existence of TSV parasitic capacitance will delay the signal transmission, so it is very important to study how to reduce the parasitic capacitance of silicon vias [24]. The calculation of parasitic capacitance of silicon via and the influence trend of the TSV physical structure on parasitic capacitance have been emphatically analyzed.

### 3.3. Development of TSV Technology

TSV is a subversive technology that is considered an effective way to achieve “beyond Moore’s Law”. With the development of micromachining technology, TSV technology is gradually maturing, but there are still many problems to pay attention to and solve on the basis of the existing research on TSV technology. The following describes the shortcomings of TSV technology development:

(1) In the future, polishing solutions for the TSV barrier layer, optimization of the polishing process, cost reduction and environmental protection will become research hotspots. With the development of the economy, science and technology in China, it is the direction for domestic researchers to gradually replace imported polishing solutions with domestic polishing solutions with the same effect;
(2) The test architecture and repair mechanism of TSV. There will be various faults, such as interconnection disconnection, short circuits, bridges, gaps, and thermal and physical stress in the process of TSV manufacturing and superposition. The manufacturing defects of TSV lead to huge yield loss in the design process of TSV before and after combination [73]. If there is no complete testing system and TSV repair mechanism, TSV failures will continue to cause huge costs due to the dumping of bad molds. Among them, the repair mechanism provides a redundancy feature, which can replace the faulty TSV with a spare TSV in the design. Compared with the standard TSV testing method, this has a significant impact on the output. Some researchers have put forward online TSV repair technology based on chain TDMA, but it is not able to repair the cluster TSV fault, which limits its applicability. In order to achieve high-yield cluster TSV failures and cost-effective hardware overheads, a new bee–TDMATSV repair method without using RTSV has emerged, thus reducing area overheads and improving outputs. Among them, cellular-TDMA is a highly reliable repair mechanism, which allocates time slots for each TSV existing in the design and provides necessary control signals to pass a good path [74];
(3) Compared with the prior achievements, the development of some new integrated circuit packaging structures and manufacturing methods can realize high-density wiring capability without forming an interlayer with the TSV; therefore, the required cost is lower;
(4) Some new chip packaging structures and preparation methods of chip packaging structures realize the direct contact of the chips on both sides by directly opening staggered slots on the adapter plate. This allows for the chips on both sides to be directly electrically connected, avoiding opening TSV through holes to realize the interconnection of the chips on both sides and effectively utilizing the bearing and electrical connection functions of the adapter plate.

By improving the structure of the interlayer, the limitation of the depth-to-width ratio in the process, such as electroplating and filling the metal layer in the TSV hole, is overcome, the deformation influence of the interlayer at high temperature is reduced, and even the independent use of the integrated circuit substrate can be omitted, thus, saving the cost and power consumption.

## 4. TCV and TGV Technology

### 4.1. Introduction of TCV

Due to ceramics’ advantages in terms of high thermal conductivity, high heat resistance, high electrical insulation, high mechanical strength, a low coefficient of thermal expansion (CTE), excellent corrosion resistance and radiation resistance, TCV is a new interconnection technology used in high-density three-dimensional packaging and maximizes the structural density in three dimensions. Under the same size, TCV technology can create more devices with good high-frequency performance, which provides a solution for micro-system power supply circuits, passive component integration and the high-density combination of multifunctional devices and units [75].

#### 4.1.1. Process Flow Design

**Figure 31:**
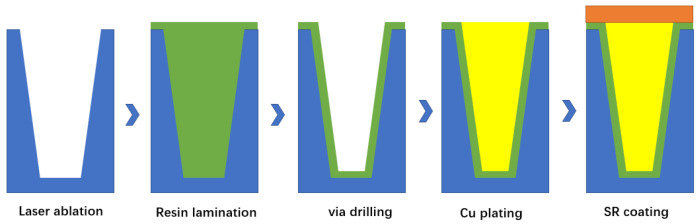
The TCV process flow chart.

The integration process of TCV has been improved and developed on the basis of the traditional thin-film manufacturing process, while common technologies such as metallization, lithography, development, corrosion, electroplating and integrated resistance are important links. The core technologies that TCV needs to pay attention to are micro-vias, full-hole metallization, multi-layer wiring, 3D ceramic stacking and so on [76]. In addition, special attention should be paid to the compatibility of the multi-process and implementation process on general technologies and key technologies. The manufacturing process of TCV is shown in Figure 31. A ceramic via is formed on a silicon substrate by laser ablation. This maskless process simplifies the preparation process of the via and does not require photolithography. Next, the dielectric resin is filled, and a thick dielectric layer is formed through the second through hole drilling. Then copper plating is carried out and SR coating is added for high-temperature wear protection, and finally, the whole surface is cleaned [76].

The 3D stacking of TCV technology is realized by connecting ceramic perforations between hierarchical substrates. The traditional thin-film process usually uses laser drilling and sputtering metallization processes to prepare hollow grounding holes. This method can not meet the requirements of flip chip solder balls and high-frequency signal transmission between TCV layers, so it is necessary to study the preparation process of solid holes for 3D ceramic connection. The following two solutions can form 3D communication holes in ceramics.

(1) Slurry filling

**Figure 32:**
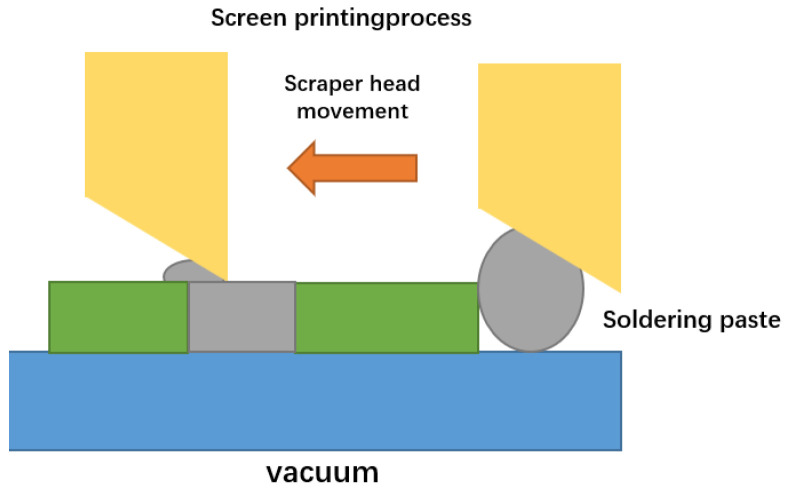
Schematic diagram of slurry printing hole filling.

Due to the consistency between the substrate and the paste, solid through holes can be formed in Al_2_O_3_, and the ceramic substrate can be filled with reference to the paste printing process. Coating a template on a ceramic substrate, coating paste in holes with a scraper, and then drying and sintering to obtain a substrate with through holes filled with slurry. The schematic diagram is shown in Figure 32 [18].

(2) Electroplating filling

Inspired by the concept of TSV, TCV can also fill the through hole by deep hole electroplating, which can achieve the advantages of good air tightness and excellent conductivity. However, this scheme also has some shortcomings because in the process of deep hole electroplating, the exchangeability of the solution in the hole is poor, and the electroplating speed on the surface of the hole is higher than that on the inner wall, so it is easy to form a mushroom-like filler or to seal it in advance to make it a hollow hole. Therefore, by optimizing the electroplating parameters and reducing the difference in electroplating speed between the hole surface and the hole, the solid hole could be filled in the study [77].

#### 4.1.2. TCV Process Optimization and Reliability

This technology is still in its infancy in China. In terms of new technologies, 3D laser processing, deep groove etching and photosensitive glass cutting could be developed and applied. In addition to ceramic substrate stacking in the material system, heterogeneous stacking of different substrates (such as glass and sapphire) can also be developed, providing increasingly effective solutions for multi-functional heterogeneous integration and micro-system integration.

Qiang Wang et al. [78] researched filling-based DC optimization. Chronoamperometry and solution conductivity measurements were used to optimize the electroplating solution. The through hole section’s electroplating effect was used to estimate process parameters such as current density and air stirring flow rate. The optimized solution included 120 g/L CuSO_4_, 55 g/L H_2_SO_4_, 50 mg/L Cl^−^, 200 mg/L PEG8000, 7 mg/L SH110 and 6 mg/L NTBC.

To optimize the thermo–mechanical reliability of the ceramic substrate with an in-line copper-filled TCV structure, Tingrui Gong et al. [75] employed the Taguchi–Grey approach and finite element modeling. On the thermal stress of TCV, the effects of six design parameters—TCV diameter, TCV height, spacing, substrate material, metal layer thickness and substrate cross-sectional area—were analyzed. For thermal stress, the order of these six factors was: the substrate material > diameter > the pitch > the metal layer thickness > the TCV height > substrate cross-sectional area. The proportion of substrate material, TCV diameter and pitch was relatively large—46.7%, 26.49% and 9.38%, respectively.

### 4.2. Introduction of TGV

The appearance of a glass-based transfer plate provides another choice for people. Glass material is an insulating material with a low loss coefficient and excellent electrical properties. In addition, when metal is filled with TGV, the sidewall does not need to be oxidized and insulated, thus reducing the production cost. Therefore, TGV technology has broad application prospects in the field of 3D MEMS packaging, especially its technical advantages of small size and no leads, which makes the TGV technology have application potential of miniaturization and high reliability in aerospace equipment [79].

#### 4.2.1. The TGV Process

**Figure 33:**
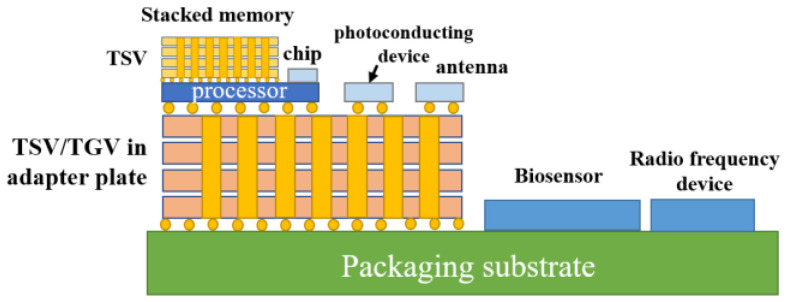
Application of TSV/TGV adapter board in a 3D integrated circuit.

TGV, that is, the glass adapter plate, punches holes in the glass and the electroplate’s copper columns to realize the communication between the upper surface and the substrate. As shown in Figure 33, it realizes the transformation from a two-dimensional planar circuit to a 3D circuit, and further promotes the integration of passive devices [80].

**Figure 34:**
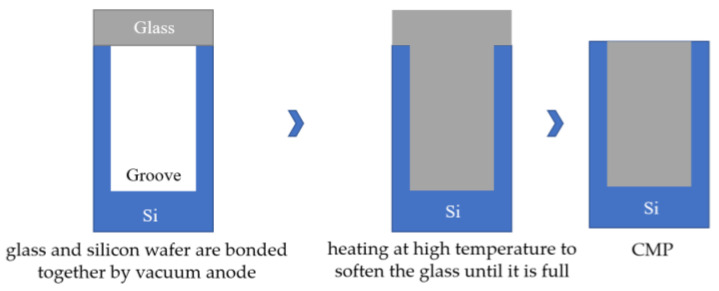
Schematic diagram of the basic manufacturing process of TGV substrate.

The silicon wafer is engraved with a deep groove with a certain depth, and the glass sheet is covered on the grooved silicon wafer surface, while the anodes are bonded together in a vacuum environment. Heat at high temperature to soften the glass and under the action of atmospheric pressure, the glass flows into the tank until it is full; double-sided CMP exposes the silicon conductive column. The silicon conductive column can realize the longitudinal interconnection of electrical signals, and the lead wire does not need to pass through the sealing ring, which can better realize vacuum sealing. The two processes shown in Figure 34 are the glass reflow process and the glass thermal reflow process; one of the new methods to realize the TGV packaging structure. Its typical process route is called glass-in-silicon reflow (GISR) [81].

**Figure 35:**
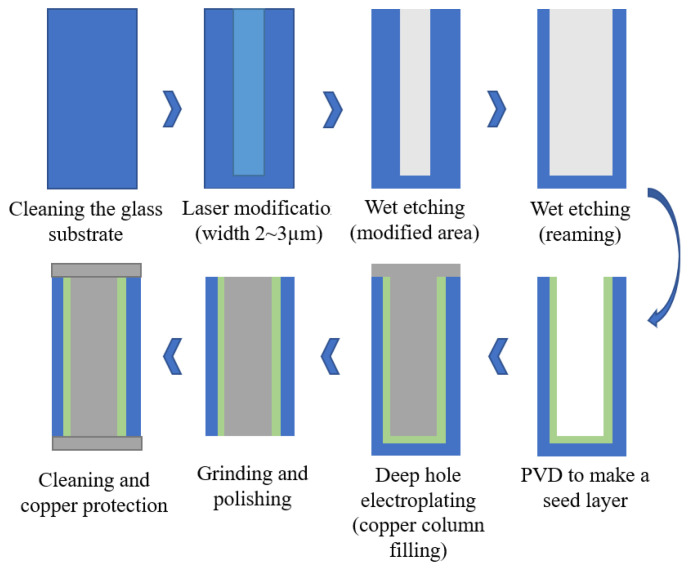
The process flow chart of high-density TGV.

At present, the research on TGV technology based on glass reflow is hot at home and abroad, while MEMS packaging schemes and products based on TGV technology are constantly proposed and realized. For example, Sensonor Technologies AS has proposed and produced a butterfly wing silicon micro-gyroscope based on glass reflow TGV packaging, and Peking University has proposed a three-layer wafer-level vacuum packaging scheme based on TGV technology, which can fill silicon deep grooves with feature sizes as small as microns. Wafer-grade borosilicate glass is widely used as a thermal reflow glass material because of its adaptive thermal expansion coefficient, good insulation and optical properties. In the GISR process, reflux temperature and holding time are the most important parameters. The process flow chart of high-density TGV is shown in Figure 35 [82] and comprises the following basic process steps:

(1) Selective laser modification is carried out on the clean glass substrate. The high-energy and high-power density laser focused inside the glass breaks the silicon–oxygen bond and forms a micro-activated region with a diameter of 2~3 μm on the glass substrate;
(2) Then, the laser-modified substrate is wet etched by using a strong alkaline etching solution with a certain concentration. In the etching process, the uniformity of the etching solution is maintained by stirring and ultrasonic wave. The heating temperature is adjusted to control the reaction speed and the etching time accurately so as to obtain a through hole;
(3) Then a seed layer with a Ti–Cu structure with good adhesion to the inner wall of the through hole and the surface of the substrate is deposited by physical vapor deposition (PVD);
(4) Copper is plated on the surface of the through hole and the substrate through the deep hole electroplating process. Considering the huge difference in the thermal expansion coefficient between the glass and copper, in order to avoid the thermal mismatch between the copper column and glass substrate under high power conditions, the high temperature of the transfer plate could lead to the fracture of the transfer plate; however, through hole electroplating is carried out by a partially hollow process. The copper column in the through hole is not completely filled, leaving gaps at both ends of the copper column, which provides space for thermal expansion of the copper column under high power conditions;
(5) Grinding the copper on the thinned surface with micron-sized grinding media: nano-alumina powder is used to polish and remove copper and crystal layer on the surface to ensure the smooth and clean surface of the substrate; Finally, clean the surface of the substrate and remove impurities (organic materials and polishing liquid residues attached to the surface), and use a copper protective agent to form a protective film on the upper and lower surfaces of the copper column to prevent the copper surface from oxidation.

#### 4.2.2. TGV Process Optimization

The main results are as follows:

(1) GISR process parameter optimization

The research on GISR technology mainly focuses on the design and implementation of the whole package scheme, and there are few documents about the details of the glass reflow process. A general model and equation considering the details of groove width, groove depth, temperature and time have not been established. Therefore, Qijun Hu et al. [81] proposed and established a general model of glass reflow by studying the effects of gap depth, gap width, temperature and time parameters on glass reflow. The relationship between the flow length and time of the glass in the microchannels under a specific groove width, groove depth and temperature was derived. Xiaohui Du et al. [83] used a nano-glass powder reflow process to prepare a TGV structure for leadless packaging so that a better nano-glass powder process and high-temperature reflow process parameters were obtained. The refluxed glass can effectively fill the large plane groove with silicon columns and the groove with high aspect ratio, and the lower corner of the groove can be completely filled without bubbles in the glass block.

(2) Optimization of TGV heat dissipation structure

**Figure 36:**
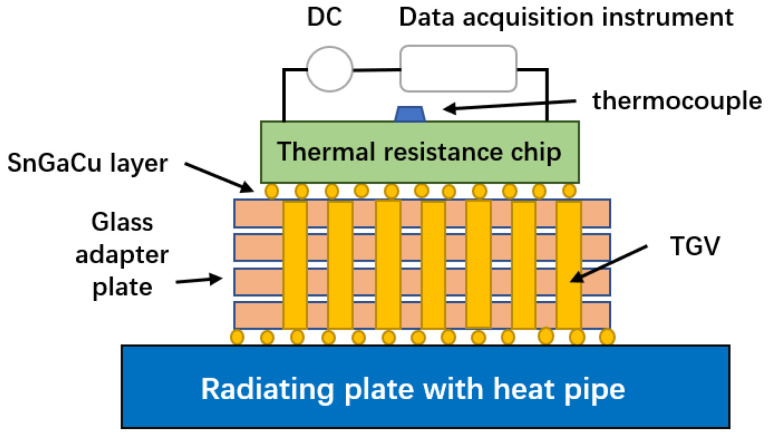
Schematic diagram of the test method for heat dissipation performance of the TGV adapter plate.

Because the thermal conductivity of the glass substrate is 0.52~1.28 W/(m·k), the low thermal conductivity finds it difficult to meet the high-efficiency heat dissipation requirements of high-power microwave devices. Therefore, it is necessary to make full use of the good heat dissipation characteristics of metallic copper, to design a high-density copper column arrangement for the glass transfer plate and form a TGV heat dissipation structure to improve the heat dissipation ability, thus, ensuring good reliability of the TGV transfer plate packaging and integration. Therefore, Qiangwen Wang et al. [82] designed and analyzed the heat dissipation structure of TGV according to the high heat dissipation performance requirements of TGV. The finite element model of the TGV adapter plate packaging integrated structure was established, the heat dissipation structure of the TGV adapter plate copper column array was designed, and the TGV high-density array was made by the TGV process, as shown in Figure 36.

(3) Optimization of TGV process parameters

**Figure 37:**
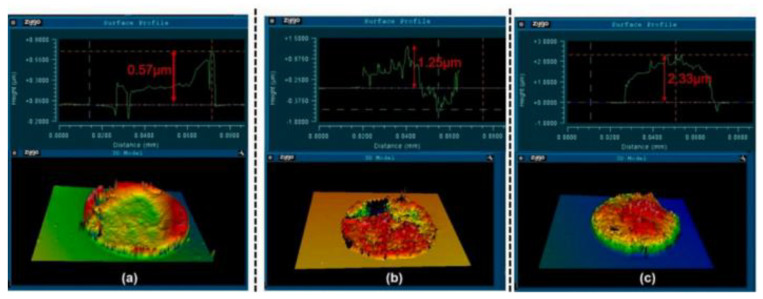
The measurement of Cu protrusion height by heating rate (**a**) 26 °C/min; (**b**) 13 °C/min; (**c**) 6.5 °C/min (adapted from [84] with permission).

There are two kinds of thermo–mechanical induced cracks in metalized TGV substrates: radial cracks and circumferential cracks. Radial cracks are formed during heating, while circumferential cracks are formed during cooling. However, radial cracks have higher reliability problems because they may lead to catastrophic failure due to the network of adjacent cracks. Therefore, Chukwudi Okoro et al. [84] understood and eliminated the formation of radial cracks. Their results showed that the number of radial cracks increased with the increase of heating rate, and the dependence of radial crack probability on the applied annealing rate followed an exponential function. When the heating rate was less than 6.5 °C/min, no cracks were formed; that is, radial cracks were eliminated. In order to study the stress relaxation activity, the functional relationship between the copper protrusion height of TGV and the annealing heating rate was measured, as shown in Figure 37. Jin Zhao et al. [85] discussed the cracks in the 3D-TGV interconnection structure through experiments and numerical simulation. The influence of geometry and material characteristics on the TGV interconnection was studied by the finite element method. Finally, some technological processes and improvement measures were put forward. The results showed that the copper protrusion decreased with the increase of the heating rate. This means that at a faster heating rate, less stress is released. However, at a lower heating rate, the large protrusion of Cu led to the reduction of the Cu volume of extruded glass substrate, thus reducing the induced substrate stress and minimizing the possibility of forming radial cracks.

(4) Optimization of the preparation process of the TGV glass seed layer

Complete filling can reduce potential risks, improve the reliability of signal transmission and reduce energy loss. Because of the low processing temperature, low processing cost and simple manufacturing and processing, chemical plating is a common method for preparing the seed layer. Yuzhe Chen et al. [86] realized a method of preparing the glass seed layer by nickel colloid activation and electroless nickel plating. In order to improve the characteristics of the seed layer, the nickel activation process was optimized comprehensively. A detailed annealing treatment was carried out to improve the adhesion of contact points. After optimization, the deposition of the Ni–P seed layer obtained good coverage and contact adhesion.

#### 4.2.3. Development of TGV Technology

Glass materials have greater potential in the miniaturization of passive devices such as filters and inductors, and TGV technology has a greater potential in chip packaging applications. The development of glass-based technology has yet to be developed, and other high-performance, miniaturized RF devices, such as AIP packaged antennas, high Q chip inductors and power splitters, can also be made by using glass substrates.

For the 3D integration process, the technical development of TGV has not been widely studied at home and abroad, and its reliability research needs to be optimized. It is the key direction of TGV 3D integration in the future to realize the complete process of glass substrate RF devices to 3D packaging through glass bonding technology.

The modeling of the TGV process is not perfect, and the simulation analysis of interlayer capacitance, contact resistance and conductivity needs to be refined. The loss caused by the process can be further improved through model analysis.

### 4.3. Process Limitation

The TCV and TGV processes are used in semiconductor packaging to create vertical interconnections through chips or substrates. However, these processes have certain limitations in terms of via diameter, via height, aspect ratio and via cavity. An explanation of these limitations is discussed below.

A larger hole diameter can provide a lower resistance and voltage drop, so it can carry a higher current. This is particularly important in high-power applications and high-frequency signal transmission. A smaller hole diameter can reduce signal crosstalk and signal losses and improve signal reliability and transmission quality. Especially in high-speed communication and microwave applications, a smaller hole diameter can reduce signal distortion and transmission delay and improve system performance.

A higher hole height will lead to larger resistance and capacitance, increase the delay and loss of signal transmission, and may also lead to uneven material filling, holes, poor filling, or material accumulation, and lead to more fragile and easily damaged interconnection structures that are prone to stress concentration and fracture. A lower hole height can reduce the resistance and capacitance, improve the speed and quality of signal transmission, and realize uniform filling and coating more easily. It can also increase the mechanical stability and strength of the interconnection structure so as to improve the stability of the process and the reliability of the products.

A comprehensive evaluation of the ratio of height to diameter shows the influence of the aspect ratio: a higher aspect ratio may lead to uneven distribution of filling and coating materials in the interconnection holes, and also lead to greater signal transmission delay and loss, which may affect the transmission quality and system performance of high-frequency signals. A higher aspect ratio may lead to more fragile interconnection structures and easy stress concentration. A lower aspect ratio not only makes it easier to achieve uniform filling and coating but also improves the mechanical strength and stability of the interconnection structure and can also reduce the crosstalk and loss of the signal and improve the signal integrity.

In practical applications, it is necessary to comprehensively consider the influence of these factors on electrical performance, manufacturing cost, signal integrity and structural stability.

## 5. Summary

### 5.1. Process Optimization

#### 5.1.1. Structural Problems

- About TSV:
  (1) The problem of micro-grass on the bottom can be solved by mask technology, while the morphology problem can be optimized by shortening the etching time, prolonging passivation time, increasing bias power and increasing the C_4_F_8_ flow rate;
  (2) After the hole DRIE, the sidewall smoothing process, that is, thermal oxidation, using 40% potassium hydroxide at 60℃, shortening the etching time and passivation time under a one-step cycle, and replacing the through etching with the combination of blind etching and back thinning, can reduce the “scallop” size of the sidewall of the TSV hole.

- About TCV:

A filled hole is formed by reducing the plating speed difference between the hole surface and the hole.

- About TGV:

A heat dissipation structure of the copper column array is added.

#### 5.1.2. Electroplating Filling

- About TSV:
  (1) Electroplating is carried out in a segmented current mode, and the electro-plating effect can be ensured by adding jitter settings;
  (2) The bottom-up electro-plating process has improved the problem of the large stress caused by excessive edge thickness due to an uneven rate;
  (3) The problem of slit defects or bulge/void defects can be solved by two-step electroplating.

- About TCV:

A DC electroplating Cu solution was produced by refining the electroplating solution formula and modifying the auxiliary process variables.

- About TGV:

Refluxed glass can effectively fill the large plane groove with silicon columns. Grooves with a high aspect ratio and the lower corner of the groove can be completely filled without bubbles in the glass block.

#### 5.1.3. Polish

- About TSV:
  (1) A step-by-step polishing process has the characteristics of a high removal rate, controllable polishing time, low risk of debris, low selectivity and few concave and convex defects.
  (2) The fine polishing of TSV can be realized by introducing the corrosion inhibitor TAZ and surfactant betaine into the glycine–H_2_O_2_ system. Adding HEDP, ozone bubbles, electro-Fenton reaction and anodic oxidation can all improve the CMP effect.

### 5.2. Reliability Thermal Stress Problem

#### 5.2.1. Structural Parameter Influence

- About TSV:

By comparing the stresses of the three shapes, it is found that the annular TSV is the least affected by the change of structural parameters, and its thermo–mechanical stability is better. The order of influencing factors on the stress of the TSV interconnection structure is: copper pillar diameter > bump material > copper pillar height > TSV spacing > bump height > bump diameter > SiO_2_ layer thickness.

- About TCV:

The thickness of the metal layer is more sensitive to thermal deformation. Reducing the cross-sectional area of the substrate is helpful in reducing thermal deformation and plastic strain, but it may increase thermal stress. Reducing the height of the TCV or the thickness of the ceramic substrate is helpful to reduce thermal deformation, but it may be unfavorable to reduce thermal stress and plastic strain.

- About TGV:

Copper protrusion decreases with the increase in heating rate. Stress increases with the increase of copper percentage in the hole, and the maximum stress value is concentrated in the hole. With the increase of RDL thickness, the maximum stress points are mainly distributed at the edges of TGV and RDL.

#### 5.2.2. Material

- About TSV:
  (1) The thermal stress of tungsten TSV in metal materials is the smallest;
  (2) Carbon nanotubes generate the least heat and can form relatively good filling materials.

- About TCV:

When the substrate material is Si_3_N_4_, thermal deformation and thermal stress are the minimum. However, the minimum plastic strain value occurs when AlN is used.

- About TGV:

Preparation of the glass seed layer by nickel colloid activation and electroless nickel plating can improve the seed layer.

#### 5.2.3. Reduce Thermal Stress

- About TSV:
  (1) The smaller the diameter of the TSV, the greater the temperature rise of the TSV silicon adapter plate. The smaller the spacing of TSVs, the greater the local heat flux density. In order to ensure electrical performance, we should, as far as is practical, use a TSV design scheme with a small size and wide spacing;
  (2) With the increase of TSV length, the Joule heat generated in TSV gradually decreases. The larger the pore size of TSV, the more obvious the influence on Joule heat, thus accelerating the failure of electromigration.

- About TCV:

Reducing the diameter of TCVs and increasing the spacing between TCVs will effectively reduce thermal stress and improve the thermo–mechanical reliability of ceramic substrates.

- About TGV:

Reducing the thermal mismatch strain between glass and copper is a method to reduce stress.

#### 5.2.4. Reduce Capacitance

- About TSV:

The depth and radius of TSV are positively related to the parasitic capacitance of the insulating layer, the thickness of the insulating layer is inversely related to the parasitic capacitance of the insulating layer, and the pitch between silicon vias is inversely related to the parasitic capacitance of the substrate.

### 5.3. Contrast

By comparing the three technologies, this paper draws the following conclusions:

(1) Compared with ceramic and organic substrates, the manufacturing technology of a silicon-based adapter board has the following advantages: it has high compatibility with semiconductor technology, greatly improves the fan-out ability and realizes the requirements of high-precision technology. The thermal expansion coefficient of silicon material is highly matched to that of silicon chips; it has good mechanical stability. However, devices of the same size have more functions and better high-frequency performance in TCV technology.
(2) Compared with TSV, glass material is an insulator material with a very small loss factor and excellent electrical properties, therefore, TGV technology has obvious advantages in high-frequency and high-speed applications. When TGV is filled with metal, the sidewall does not need to be oxidized and insulated, thus reducing the production cost. TGV has technical advantages in small volume and leadless packaging.

## Figures and Tables

**Table 1:** The process parameters for the best effect of blind hole electroplating.

	Current Density (A/m^2^)	Current (A)	Time (s)
The first paragraph	0	0	300
The second paragraph	0.08	0.24	120
The third paragraph	0.16	0.48	7200
The Fourth paragraph	0.18	0.54	1800
Other conditions	Vacuum pretreatment: −90 KPa; Vibration frequency: 20 Hz		

**Table 2:** TSV CMP process parameters.

Silicon via Filling Material	Deposition Thickness/nm	Planarization Rate/(nm·min^−1^)	Disc Depression/nm
copper	500∼60,000	100∼8000	1~300
polysilicon	400∼3000	200∼1500	30~120
wolfram	300∼900	300∼800	15~30
platinum	1500∼5000	150∼500	10~80

## Data Availability

Not applicable.
